# Decoding rule search domain in the left inferior frontal gyrus

**DOI:** 10.1371/journal.pone.0194054

**Published:** 2018-03-16

**Authors:** Michele Furlan, Laura Babcock, Antonino Vallesi

**Affiliations:** 1 Department of Neuroscience, University of Padua, Padua, Italy; 2 Department of Neuroscience, Karolinska Institutet, Stockholm, Sweden; 3 IRCCS San Camillo Hospital Foundation, Lido-Venice, Italy; Universita degli Studi di Udine, ITALY

## Abstract

Traditionally, the left hemisphere has been thought to extract mainly verbal patterns of information, but recent evidence has shown that the left Inferior Frontal Gyrus (IFG) is active during inductive reasoning in both the verbal and spatial domains. We aimed to understand whether the left IFG supports inductive reasoning in a domain-specific or domain-general fashion. To do this we used Multi-Voxel Pattern Analysis to decode the representation of domain during a rule search task. Thirteen participants were asked to extract the rule underlying streams of letters presented in different spatial locations. Each rule was either verbal (letters forming words) or spatial (positions forming geometric figures). Our results show that domain was decodable in the left prefrontal cortex, suggesting that this region represents domain-specific information, rather than processes common to the two domains. A replication study with the same participants tested two years later confirmed these findings, though the individual representations changed, providing evidence for the flexible nature of representations. This study extends our knowledge on the neural basis of goal-directed behaviors and on how information relevant for rule extraction is flexibly mapped in the prefrontal cortex.

## Introduction

A recent body of evidence suggests that executive functions might be subject to a degree of functional fractionation and that they can be mapped along the axes of the prefrontal cortex (PFC) [1–5]. This fractionation might extend to the left-right dimension, implying hemispheric specialization, or at least gradients of organization, in supporting executive functions. However, the way executive functions are mapped along this dimension still needs to be fully understood [6]. One central issue is to assess whether the fractionation along the left-right dimension is domain-based, process-based or a combination of the two.

On one side, some studies suggest that the fractionation along the left-right dimension is mostly domain-based. Evidence from neuroimaging studies shows that verbal information is preferentially processed in the left ventrolateral PFC (vlPFC), while spatial information is preferentially processed in the right vlPFC [7,8], suggesting that hemispheric specialization is domain-based.

However, some recent models [9,10] propose that the fractionation along the left-right dimension is process-based. Particularly interesting is the Goel’s model describing the reasoning abilities of humans [10]. This model suggests that the left PFC has a role in reducing uncertainty by processing conceptual connections and logical relations among the available information, while the right PFC has a role in maintaining and even enhancing uncertainty by overcoming preconceptions based on prior beliefs and therefore allowing us to explore a wider range of possible solutions. These cognitive mechanisms are particularly important for our inductive reasoning abilities. Indeed, inductive reasoning is a complex function that allows us to increase our understanding of the world by making predictions based on our previous knowledge [11]. Although the complex set of computations underlying inductive reasoning is carried out in a large network of brain regions, a central role is played by the left PFC. In fact, this brain structure has been shown to be particularly sensitive to rule induction [12–14] and rule checking [15]. These studies support the hypothesis that hemispheric specialization is process-based rather than domain-based. Viewed together with the studies mentioned above, it seems that the organizing principles underlying the lateralization of executive functions are still unclear. To explore these principles, we employed an inductive reasoning task.

Inductive reasoning was used recently to address the question of whether the gradient along the left-right dimension is domain-based by manipulating rule domain within different types of rule search [16]. Babcock and Vallesi (2015) measured the brain activity when participants were engaged in extrapolating rules based on verbal or spatial information. Extracting rules recruited a large network of regions, among which the largest activations were found bilaterally in the Inferior Frontal Gyrus (IFG), in the left Superior Frontal Gyrus (SFG), bilaterally in the lateral Orbital Gyrus (lOG) and in a portion of the left cerebellum. Interestingly, the right IFG was activated only when extracting spatial rules, suggesting a selective preference for spatial information. On the other hand, extracting both verbal and spatial rules activated a common region in left IFG, as confirmed by a conjunction analysis, suggesting that the left IFG is involved in rule extraction in both domains.

The latter evidence is particularly intriguing, and supports at least two possible explanations. The first is that the left IFG supports a domain-independent cognitive process required for the general task, such as rule extraction. The second possible account is that the left IFG holds representations of domain-specific information required for the task. These two possibilities could not be disentangled using the univariate analyses presented in Babcock and Vallesi (2015). Multivariate analyses, however, offer an opportunity to support one or the other explanation as these analyses are intended to specify the type of information processed in a region. Indeed, Multi-Voxel Pattern Analysis (MVPA), a type of multivariate analysis, is sensitive to information contained in fine-grained patterns, which goes undetected by univariate analysis [17,18].

These fine-grained patterns can separate the two posited explanations. For the first, we expect that the left IFG is employed in the same manner for both domains, thus there would be no difference in the neural patterns associated with the verbal and the spatial domains in this region. For the second explanation, we expect that the left IFG holds independent representations for the verbal and the spatial domains. These representations should be decodable in this region as separate neural patterns.

To tease apart these two explanations we employed a support vector machine (SVM)-based MVPA to examine whether rule domain can be decoded in the left IFG. Specifically, we examined the representations of the verbal and spatial domains in the regions involved in the rule discrimination phase as reported by Babcock and Vallesi [16]. The ability to decode domain in the left IFG in our data would support a domain-specific function of this region, while the inability to do so might suggest a domain-general function. Additionally, we examined the replicability and stability of the neural representations over two years employing a longitudinal design. Replicability was addressed by considering the two time points separately, with the second one viewed as a confirmation of the first. Stability was addressed in a cross-session analysis, in which we trained the classifier on data from one time point and tested it on data from the other time point.

## Method

### Dataset

We analyzed data from thirteen participants who took part in a longitudinal study composed of two sessions (10 female, average age: 22.4±0.6 years at the first session). All participants from Babcock and Vallesi [16] were invited to participate in a second session two years after the first one; the participants who were able to return (13/20) make up the sample examined in the present study. To ensure that the returners did not represent a biased sample, we compared behavioral performance at the first session between the returners and non-returners and found no differences (*p*s ≥ .112). The two sessions used the same experimental procedure and parallel forms of same task (counterbalanced across participants). All participants were right-handed native Italian speakers with no known neurological or psychiatric conditions. All of them had normal color vision as measured by the Ishihara Color Test [19]. They were screened for MRI contraindication according to standard procedures and written consent was obtained. The study was approved by the ethical committees of “Istituto IRCCS E. Medea—La Nostra Famiglia” and Scuola Internazionale Superiore di Studi Avanzati (SISSA). The task and data acquisition procedures in both sessions were the same as those reported in Babcock et al. [16].

### Task design

**Fig 1:**
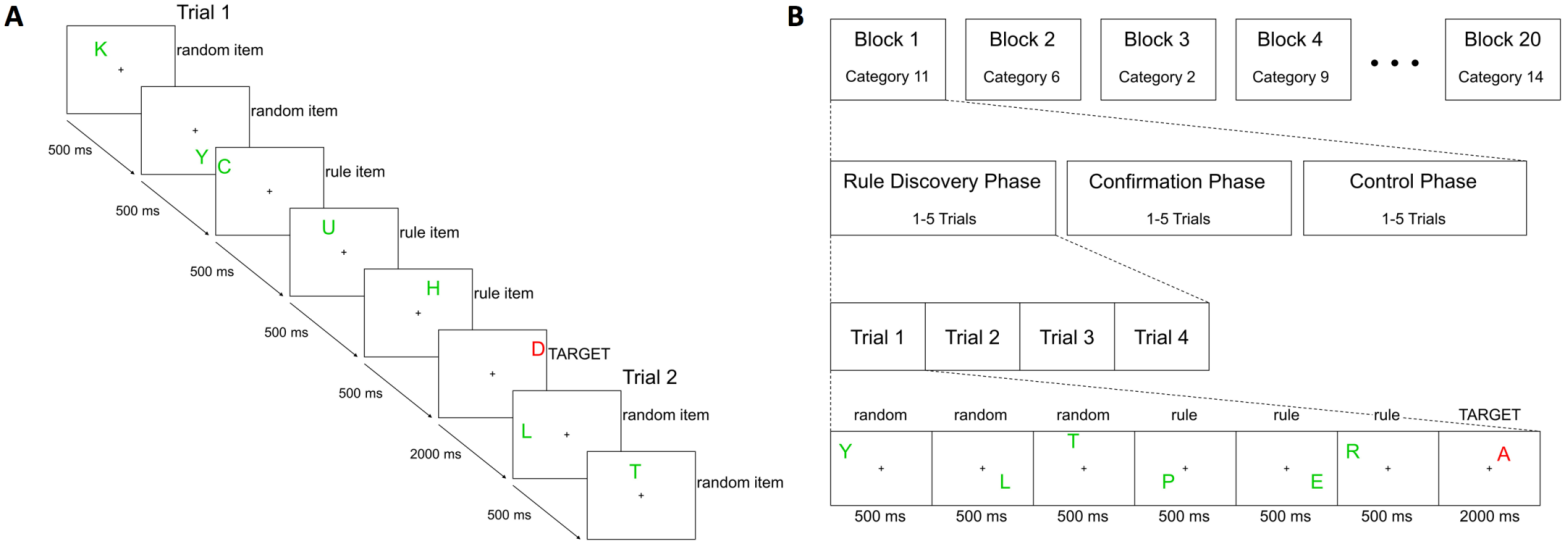
A. Example of items during rule discovery and confirmation phases. A sequence of letters was presented on the screen. The rule items followed either a spatial rule (e.g., a horizontal line) or a verbal rule (e.g., fruits). The appearance of a red letter indicated the end of the current trial. In this example, the rule was a horizontal line equally spaced from one edge to the other. B. Overview of the task structure. In this example, the rule category is fruits and the exemplar is “PERA” (pear in Italian).

Participants performed an inductive reasoning task using verbal and spatial information at each session. The task was composed of twenty blocks, each containing three phases. These phases followed the same basic procedure, but differed in the particular processes required. In each phase, participants viewed a series of capital letters (the 21 letters of the Italian alphabet) one at a time in varied spatial locations within a rectangle onscreen. These items (i.e., the letters) were grouped into trials consisting of six to twenty items. The final item of each trial was colored red (rather than green) to indicate that the trial had ended and that the participant needed to make a choice response. Additionally, the final item of each trial was presented for 2000 ms (rather than 500 ms). Following presentation of this item, a new trial began (see Fig 1A). The three phases were distinguished by the composition of the trials and the choice response required.

The first phase focused on rule discovery in the verbal and spatial domains. Note that this phase was referred to as “pattern discovery” in Babcock and Vallesi (2015), but has been renamed here to avoid confusion with the term “pattern” in the MVPA sense. The trials in this phase consisted of two to twelve random (filler) items followed by a sequence of four to eight rule items. For the random items, the letter identity and spatial location were randomly selected, as was the number of random items in each trial. These were followed by the rule items, which formed an exemplar from a category in either the verbal domain or the spatial domain. Rules in the verbal domain represented semantic categories (e.g., sports, clothing items, see the S1 Appendix for a full list) while rules in the spatial domain represented geometric figures or designs (e.g., vertical line, a semi-circle, see the S1 Appendix for a full list). Each rule category comprised ten exemplars, two of each length (4–8 items). For both domains, “rule” denotes a general category, while “exemplar” denotes a specific sequence of letters or positions within a rule category. For example, the verbal rule category “clothing items” included the exemplars gonna (skirt), giacca (jacket), and cappello (hat), among others. In the spatial domain, the general category “vertical line” indicated that the items were presented at a single x-value starting at one edge of the rectangle and proceeding in equally spaced steps to the opposite edge, while an exemplar of this category was five items at x = 135 starting at the top edge. In all categories, the exemplars were carefully selected so that the arrival of the final item of each exemplar was fully predictable once the rule was acquired, that is, there were no “catch” or misleading exemplars. For verbal rules, the specific exemplar determined the letter identity of each rule item, while the spatial location was randomly selected. Conversely, for the spatial rules, the specific exemplar determined the spatial location of the rule items and the letter identity was randomly selected. For half of the rules, the domain of the rule, that is, verbal or spatial, was indicated at the start of the phase, thus allowing participants to focus their attention on information pertaining to the relevant domain. For the other half of rules, participants were told the rule could be from either domain. In this case they needed to search both domains in parallel. The participants were aware of the composition of the items, that is, that random items would be followed by an exemplar belonging to a general category and that the end of the sequence would be denoted with a red item. They were asked to discover the rule category based on the exemplars presented and indicate whether or not they had discovered the rule at the end of each exemplar through a button press on the red item (specific response buttons were counterbalanced across participants). Once the participant indicated s/he had discovered the rule, the first phase ended and the second phase, described below, began. Alternatively, if the participant did not discover the rule after five exemplars, the participant was told the category and then a new rule discovery phase with a different rule category began.

In the second phase new exemplars from the same rule were presented and participants were asked to predict, through a button press, when the final item would arrive. This phase was used to confirm that the participant had correctly inferred the rule in the first phase. This phase included the same number of trials as its associated rule discovery phase and was followed in all cases by the third and final phase.

In the final phase, participants were asked to complete a modified version of a 1-back task designed to control for the working memory demands of the first phase (i.e., the rule discovery phase). No rules were presented in this phase; instead each trial contained six to twenty randomly selected letters at randomly selected locations. The final item of each trial was red and required a choice response. When the domain of the associated rule discovery phase was verbal, participants pressed one button if the target and the preceding item were both consonants and the other button if at least one of these two items was a vowel (specific response buttons were counterbalanced across participants). Alternatively, if a spatial rule was presented in the associated rule discovery phase, participants pressed the right button if the target and preceding item were both presented in the right half of the rectangle and the left button if at least one of these two items was in the left half (instructions with the opposite sides were given to half of the participants). As the arrival of the final item could not be predicted, participants had to continuously update the relevant information. This cognitive process resembled the working memory requirements of the rule discovery phase. This phase consisted of the same number of trials as the associated rule discovery phase.

The full task consisted of twenty blocks, which each contained a rule discovery phase, a confirmation phase that used the same rule category, and a working memory control phase that used the same domain (see Fig 1B for an overview of the entire task structure). The twenty blocks were presented across four acquisition runs. Within a run, a new block started immediately after the previous one finished. Prior to each phase in every block, a reminder of the specific task and how to respond to the target items was given after a blank screen of jittered length (2000–8000 ms in 250 ms intervals). A total of forty rules were used in the task, with each participant viewing half of the rules in the first session and the other half in the second session (counterbalanced across participants). In each session, half of the rules were from the verbal domain and half were from the spatial domain. Further half of the rules in each domain were run under separate search conditions and half under parallel search conditions.

### Data analysis

The fMRI data were first analyzed using Statistical Parametric Mapping 8 (SPM8, Wellcome Department of Cognitive Neurology, UCL, London, UK). The preprocessing of the data and the univariate analyses were carried out as reported in Babcock et al. [16]. The preprocessed data were analyzed within the General Linear Model (GLM), separately for each participant. Data were modeled using a total of nine conditions as regressors. Four of these conditions were the experimental rule discovery conditions (spatial rule-separate search, verbal rule-separate search, spatial rule-parallel search, verbal rule-parallel search), two were confirmation (phase two) conditions (spatial, verbal) and two were working memory control conditions (spatial, verbal). The final condition comprised instances of absent or incorrect rule discovery. Each condition consisted of a series of epochs, defined as a given phase (rule discovery phase, confirmation phase, control phase) for a particular rule. The duration of each epoch was determined by the onset of the first item (i.e., a letter) and the offset of the last item, with each epoch lasting an average of 17 seconds in the first session and 10 seconds in the second one. Each epoch was modeled as a box-car function convolved with the canonical Hemodynamic Response Function (HRF) [20]. Head motion regressors were included as well. An additional set of four regressors, one per run, was included in the analysis in order to model unspecific and sustained differences across the runs. We then extracted a t-contrast map for each condition of interest, separately for each participant. The resulting individual contrast maps were then submitted to a random-effects analysis, carried out using a full factorial Analysis of Variance (ANOVA) with one factor of eight levels (conditions of interest). This procedure was used to analyze the data acquired during both the first and the second session.

### ROI definition

Given that we were specifically interested in examining which processes underline the activation found in Babcock and Vallesi (2015), a univariate analysis on the data acquired during the first session was used to define the ROIs. We included all the resulting ROIs, not just the left IFG, in our examination in order to have some regions of comparison. This also allowed us to examine the homologous right IFG, which could be of particular interest given the uncertainty surrounding the left-right axis. To extract these ROIs, we used a simple t-contrast between the four rule discovery conditions (each weighted +1) and the two control conditions (each weighted -2). The statistical significance of the resulting map was set at peak-wise p < 0.001, and then corrected for multiple comparisons using a Family-Wise Error (FWE) correction (p < 0.05) at the cluster-level. Additionally, clusters with fewer than 25 voxels were discarded, as multivariate analysis requires a larger number of features (voxels) to be efficient [21]. The anatomical locations of the resulting clusters were found using the neuromorphometric toolbox provided in SPM12. These ROIs were used to carry out the ROI analysis in both the first and the second session.

### Region of interest MVPA

Multi-voxel pattern analysis (MVPA) was performed using a Support Vector Machine (SVM) with a linear kernel [22] as implemented in the CoSMo MVPA toolbox [23]. Further statistical analyses on the resulting classification accuracies were performed using R (R Foundation for Statistical Computing).

The multivariate analysis was based on voxels consisting of β weights from the GLM analysis conducted as described above. For each ROI and for each session, the univariate analysis provided us with a series of β weights that were calculated separately for each condition (8) and for each run (4), resulting in thirty-two β weights per session. These thirty-two β weights were associated both with conditions of interest (i.e., the four rule discovery conditions) and with conditions that were irrelevant to the aim of the present study (i.e., the confirmation and control conditions). Since we were interested in decoding the domain for each rule discovery condition, we submitted to the MVPA only the β weights associated with the four conditions of interest. This procedure resulted in a total of sixteen multi-voxel β patterns per subject (four conditions by four runs). This dataset was then divided in two subsets, one for each search type, resulting in eight multi-voxel β patterns associated with the separate search conditions and eight multi-voxel β patterns associated with the parallel search conditions.

**Fig 2:**
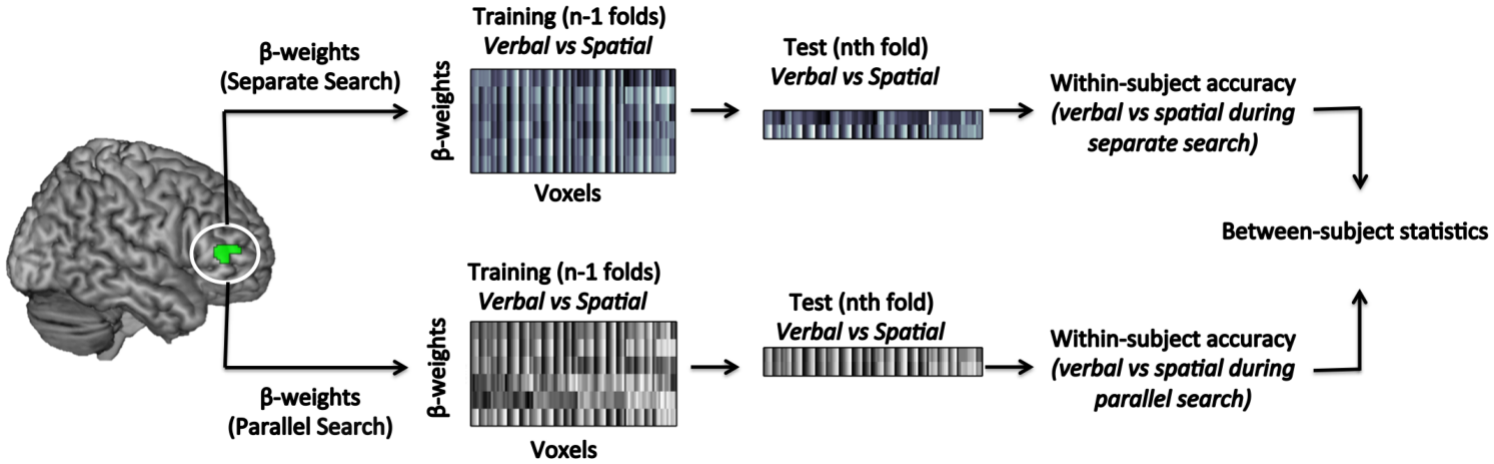
Summary of the ROI MVPA. ROIs were selected based on the contrast between the 4 rule discovery conditions (weighted +1 each) and the two control conditions (weighted -2 each). All the resulting statistical maps were corrected for multiple comparisons at the cluster level (p_(corr)_<0.05). The matrices of β values extracted from each ROI were partitioned in terms of rule search type, forming one multi-voxel β pattern for each search type (one for the separate search and one for the parallel search). The resulting multi-voxel β patterns were submitted to MVPA employing the standard leave-one-out cross-validation technique. Classification accuracies were then used in group-level statistical analyses.

The resulting β weights were then used as input for MVPA. Here, classification accuracies were computed using leave-one-out cross-validation. Specifically, for each search type, three runs were used for training and the fourth was used for testing. This procedure was repeated four times, leaving out each run in turn; performance on the testing run was averaged across the four trials. A summary of the analysis employed here is shown in Fig 2.

In order to validate whether the performance of our classifier exceeded chance performance, we carried out permutation-based statistical testing. This process allowed us to generate a null distribution that was used to examine whether a given classification performance was significant with α < 0.05. Specifically, the null distribution was calculated from the same dataset after having randomly permuted the labels, an operation that should produce chance-level accuracies with a similar variance to the main analysis. This analysis was then repeated 1000 times with different random permutations, using the same leave-one-out method, providing us with 1000 performance estimates. The 95th percentile of the estimated distribution of permuted performance results was taken as the critical value for determining significance among the experimental performance values.

To address our primary question, we ran multivariate analyses on each session separately (i.e., the classifier was trained and tested only on data from only session 1 or only session 2) to test whether rule domain could be decoded. To address the replicability of the results, we contrasted the pattern of results between the two sessions. Additionally, to explore potential differences in terms of the decoding accuracy across the left-right axis, the vectors of accuracies associated with the left and right IFG were submitted to a two-way ANOVA for repeated measures, with “hemisphere” (2) and “session” (2) as factors, conducted separately for each search type. To examine the stability of the representations of stimulus domain over time, two cross-session analyses were conducted. Specifically, a classifier was first trained on all runs from session 1 and then tested on all runs from session 2, and vice versa.

Finally, to address a possible confound represented by the number of selected features, decoding performance was examined for both the left and the right IFG as a function of the number of features (i.e., voxels) included in the analysis. This was important because the selected ROIs varied in size and thus significant MVPA results could be inflated by the size of the ROI. This bias may be introduced by the specific mechanics of the SVM. SVMs are usually trained to identify the optimal separating boundary (hyperplane) between two classes of stimuli. During this process, the *d* dimensional input vector (x) is transformed into a higher *d*^*i*^ dimensional feature vector (z) through a mapping function (z = Φ(x)), so that the new training data can be separable by a hyperplane [24]. Theoretically, it follows that a high enough dimensional space (*d’>>d*) might eventually lead the data to become separable. This is one of the possible pitfalls of SVM applied to neuroimaging data, as it suggests that the probability to find the optimal hyperplane increases with the number of voxels analyzed, resulting in an inflated decoding performance. This inflation can be reduced, if not avoided, by reducing the feature space (dimensionality reduction) through feature selection [25]. This problem is particularly relevant in MVPA studies, in which informative features (voxels) need to be wisely selected in order to make the classification task feasible [26].

A fairly neutral way to perform feature selection is to measure the decoding performance on a subset of randomly selected features. Moreover, to better appreciate how the classifier accuracy changes as a function of the number of selected features, this selection process is repeated progressively including more features; this method has been successfully used before in studies where MVPA was applied to functional data [21,27–30].

In the present study, we followed this example and analyses were run on ten samples sizes, from ten voxels to one hundred voxels, with a step size of ten, for each ROI. For each sample size, the analysis was repeated ten times with a different random selection of voxels and the resulting decoding performances were averaged. This analysis was repeated separately for each participant, and the resulting accuracy performances were averaged across participants.

## Results

### Behavioral results

The primary behavioral measure of interest was the number of rule exemplars needed for participants to indicate that they had discovered the rule. This measure can be viewed as a proxy for difficulty, with higher difficulty resulting in a higher number of exemplars. In the first session participants required an average of 2.1 exemplars to discover the rules, while in the second session only 1.3 exemplars were needed. This decrease was significant (*t*_(12)_ = 4.069, *p* = 0.002). As no rules were repeated between the sessions, this decrease likely demonstrates that participants benefitted from their previous experience with the task generally at the second session. The number of exemplars needed did not differ between the spatial and verbal domains at either session (first session: spatial = 2.2, verbal = 2.0, *t*_(12)_ = 1.190, *p* = 0.257; second session: spatial = 1.4, verbal = 1.1, *t*_(12)_ = 1.678, *p* = 0.119), suggesting that the two domains were comparable in difficulty. Comparisons between the search types showed a significant difference at the first session (separate = 2.0, parallel = 2.3, *t*_(12)_ = 3.638, *p* = 0.003) and a slight trend at the second session (separate = 1.2, parallel = 1.3, *t*_(12)_ = 2.006, *p* = 0.068) with parallel searches requiring more presentations.

### Univariate fMRI analysis

**Fig 3:**
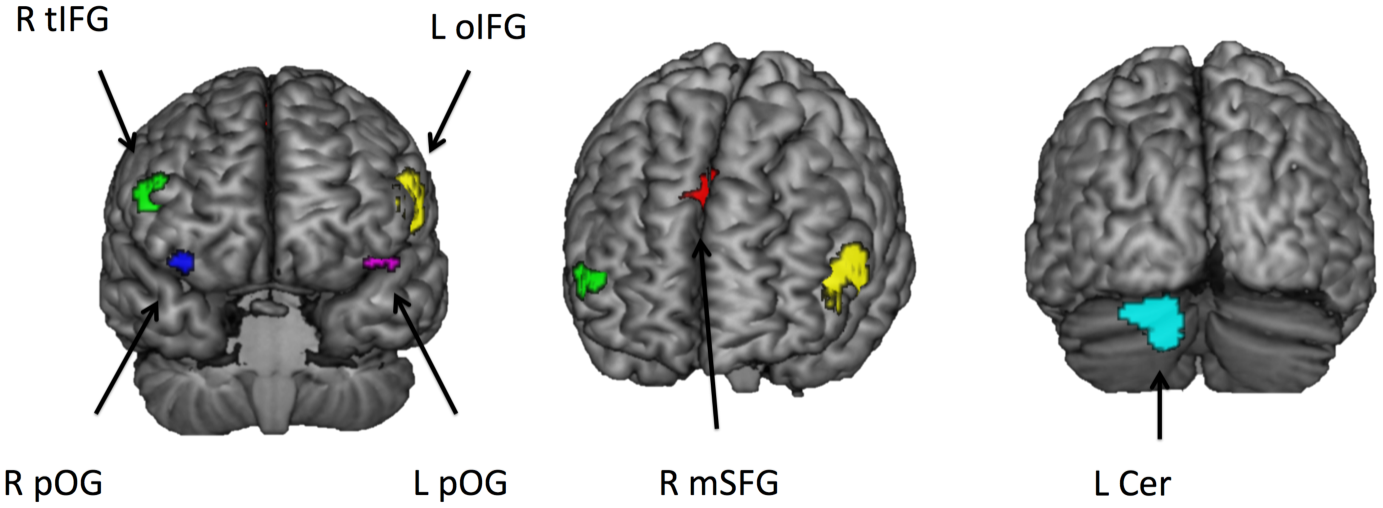
Rendered template brain in MNI space showing the position of the ROIs identified by the univariate analysis and used later in the multivariate analyses. The ROIs identified correspond to right inferior frontal gyrus (R IFG), left inferior frontal gyrus (L IFG), bilateral orbital gyrus (R and L OG), right superior frontal gyrus (R SFG), and left cerebellum (L Cer).

The ROIs used in the MVPA were localized using a group contrast between the four rule discovery conditions versus the two control conditions (for further details, see the section on ROI definition). This contrast revealed activations in the right inferior frontal gyrus (R IFG), the left inferior frontal gyrus (L IFG), bilateral orbital gyrus (R and L OG), right superior frontal gyrus (R SFG), and left cerebellum (L Cer). Fig 3 shows the position of the regions used as ROIs; the peak MNI coordinates for each ROI are reported in Table 1

**Table 1:** Clusters of activation identified by the rule discovery vs control contrast.

Anatomical Localization	BA	MNI Coordinates			Cluster p-corr	Peak z-va	Voxels per cluster
		X	Y	Z			
Left IFG	44	-56–52–56	16 35 24	10 5 18	<.0001	6.72 6.62 6.60	463
Right IFG	46	54 42 44	38 35 30	8 12 6	<.0001	7.65 5.71 5.17	167
Right SFG	8	10	32	44	<.0001	7.16	102
Left OG	47	-44–50–38	26 18 38	-12–14–18	0.023	5.73 5.69 5.53	32
Right OG	47	36	32	-26	0.032	7.31	27
Left cerebellum	/	-14–24–14	-92–86–86	-24–26–24	0.023	6.85 5.82 5.50	300

### Region of interest MVPA

**Fig 4:**
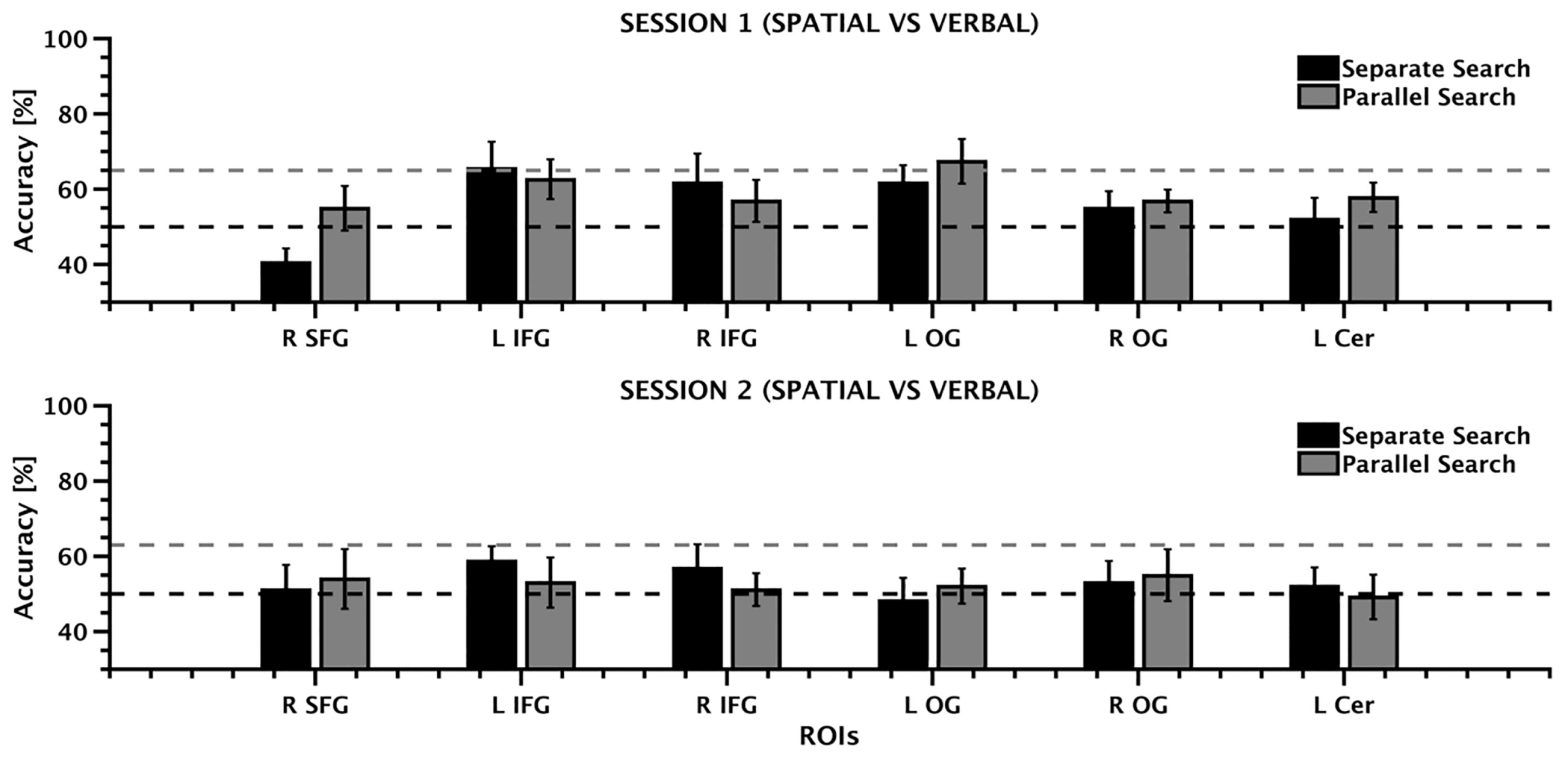
A. ROI MVPA intra session results. Mean classification accuracy for the two types of search (separate, parallel) shown separately for each ROI and session (first session: upper panel; second session: lower panel). Error bars depict ±SEM. The black line indicates the chance level (50%), while the red dashed line indicates the decoding level that is significantly above chance (>95^th^ percentile) derived by permutation testing.

The ROI MVPA was used to test the hypothesis that the regions recruited during rule discovery hold the representations of the rule domain. To this end, rule domain was decoded separately for the two search types (separate and parallel) as well as for the two sessions. The results are shown in Fig 4 while Table 2 reports the classification performance for each ROI and for each session.

**Table 2:** The mean and the standard error mean of the classification performance are reported separately for each ROI, for each session and for each search type. The 95^th^ percentile calculated from the permutation testing for each session is reported in parentheses.

	First session (95^th^: 65%)		Second session (95^th^: 63%)	
	Separate Search	Parallel Search	Separate Search	Parallel Search
R SFG	40.38±4.04	54.80±5.92	50.96±6.85	53.84±7.92
L IFG	65.38±7.37	62.50±5.29	58.65±4.09	52.88±6.66
R IFG	61.53±8.06	56.73±5.59	56.73±6.58	50.96±4.35
L OG	61.53±4.99	67.30±5.92	48.07±6.30	51.92±4.66
R OG	54.80±4.80	56.73±3.04	52.88±6.03	54.80±6.86
L Cer	51.92±5.97	57.69±3.88	51.92±5.02	49.03±5.91

**Fig 5:**
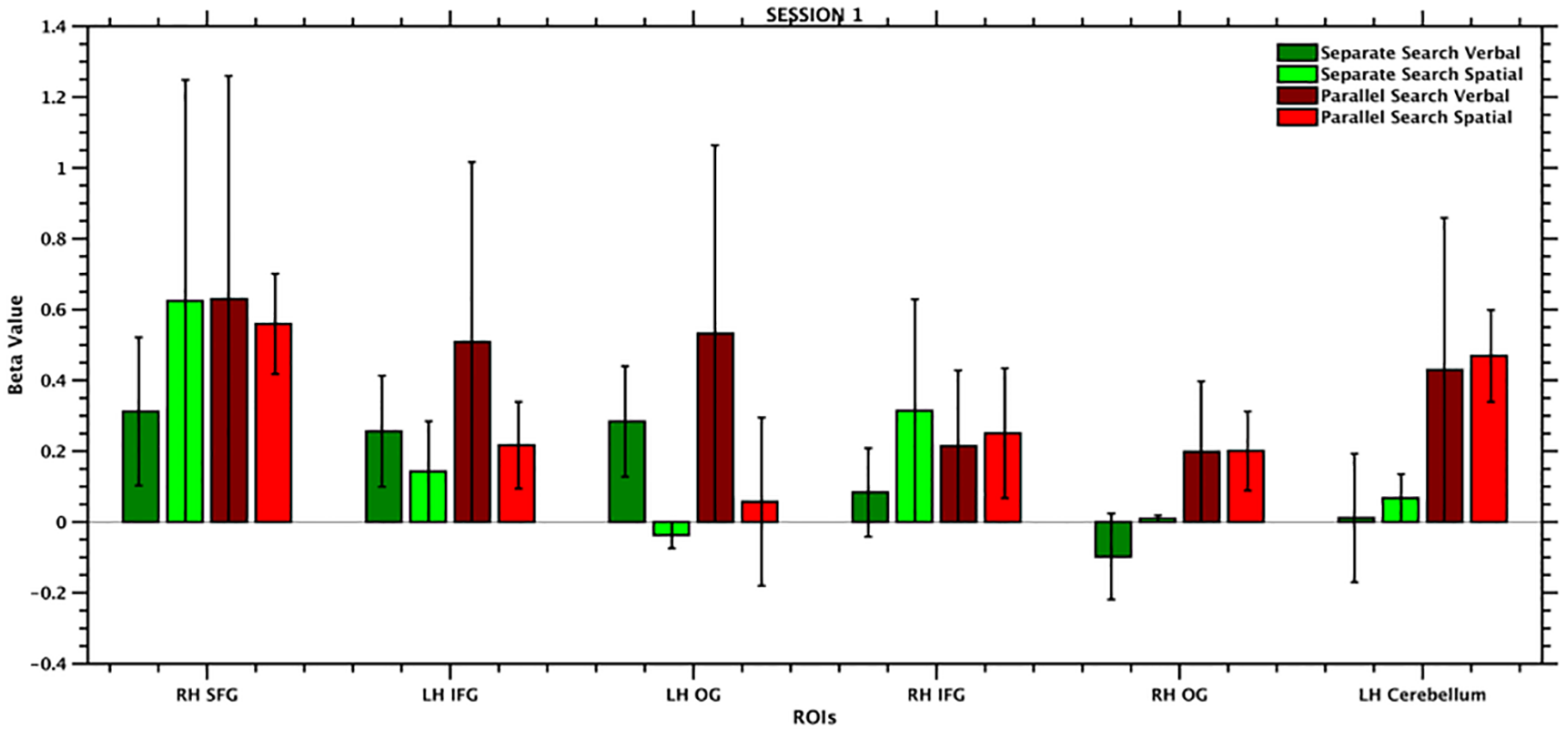
Beta values shown for each of the selected ROIs, separately for each condition. The largest difference in terms of beta values among the conditions is shown by the left OG. This difference could introduce a bias in the classification accuracy, explaining the significant results observed in the left OG.

This analysis revealed that the representation of the rule domain could be decoded in several brain regions. When the analysis was run on the first session, decoding rule domain during separate search produced above chance accuracies (i.e., 50%) in the left oIFG, in the right IFG and in bilateral OG, while decoding rule domain during parallel search produced above chance accuracies in all the regions taken in exam. When the analysis was run on the second session, we observed a different pattern, with the classification accuracy obtained while decoding rule domain during separate search being above chance in the left IFG and in the right tIFG, while the classification accuracy obtained while decoding rule domain during parallel search was above chance in the right SFG, the left IFG, in the right IFG, and in bilateral OG (see Fig 5). Although classification performance in these brain regions was above chance level, accuracy was higher than the 95^th^ percentile based on permutation testing only in the left IFG and in the left OG during the separate search condition in the first session (65.38% and 67.30%, respectively). No other brain region showed accuracy higher than the 95^th^ percentile.

Such a high classification accuracy could be generated by a genuine spatial structure or by a difference in beta values. While the former is a genuine result, the latter is a potential confound. In fact, decoding performance is known to be strongly dependent on the magnitude of the signal being decoded [31]. It follows that a given response selectivity in a given cortical region might be detected with MVPA more readily if the response magnitude elicited by the stimulus employed or by the cognitive process engaged is large than if it is small. To test the hypothesis that our classification accuracy could be generated by difference in beta value, we measured the average beta value for each ROI and for each condition and checked whether any difference in terms of beta value could explain our results. The results are reported in Fig 5 and in Table 3. As hypothesized, the largest difference resulted from the comparison between the verbal and the spatial conditions in the parallel search in the left OG (absolute difference: 0.47). This difference was by far the largest one with respect to the differences observed in the other regions (absolute differences included between 0.002 and 0.31). Among these, the difference between the verbal and the spatial conditions in the separate search in the left IFG was one of the smallest ones (absolute difference: 0.11). This pattern suggests that the results observed in the left OG could most likely be spurious and resulting from a difference in beta values between the conditions analyzed, while the results in the left IFG could not be accounted for by differences in beta values. Normalizing the beta value could easily solve this problem, although another school of thought argues that normalizing the beta value can introduce a bias in the analyses [32].

**Table 3:** Average beta value reported for each ROI and for each condition in terms of mean and standard deviation.

	Separate Search Verbal	Separate Search Spatial	Parallel Search Verbal	Parallel Search Spatial
R SFG	0.31±0.20	0.62±0.62	0.62±0.62	0.55±0.14
L IFG	0.25±0.15	0.14±0.14	0.50±0.50	0.21±0.12
L OG	0.28±0.15	-0.03±0.03	0.53±0.53	0.05±0.23
R IFG	0.08±0.12	0.31±0.31	0.21±0.21	0.25±0.18
R OG	-0.09±0.12	0.01±0.01	0.19±0.19	0.20±0.11
L Cer	0.01±0.18	0.06±0.06	0.42±0.42	0.46±0.12

In the following analysis, the factor ‘hemisphere’ will not refer to the entire hemisphere, but only to the left and the right IFG. The statistical analysis run on the classifier performance associated with separate search revealed that the classifier ability to predict the domain was the same for both hemispheres (main effect of “hemisphere”: *F*_(1,12)_ = 0.249, *p* = 0.62). The performance of the classifier across sessions was steady (main effect of ‘session’: *F*_(1,12)_ = 0.649, *p* = 0.436). Finally, the effect of the hemisphere on the classifier performance was independent of the session (two-way ‘session’ x ‘hemisphere’ interaction: *F*_(1,12)_ = 0.33, *p* = 0.859). In the analysis on the performance accuracies associated with the parallel search, the classifier distinguished each class with the same accuracies in both hemispheres (main effect of “hemisphere”: *F*_(1,12)_ = 0.766, *p* = 0.39) and in both sessions (main effect of “session”: _(1,12)_ = 1.871, *p* = 0.20), with no interaction between the variables (*F*_(1,12)_ = 0.203, *p* = 0.66).

**Fig 6:**
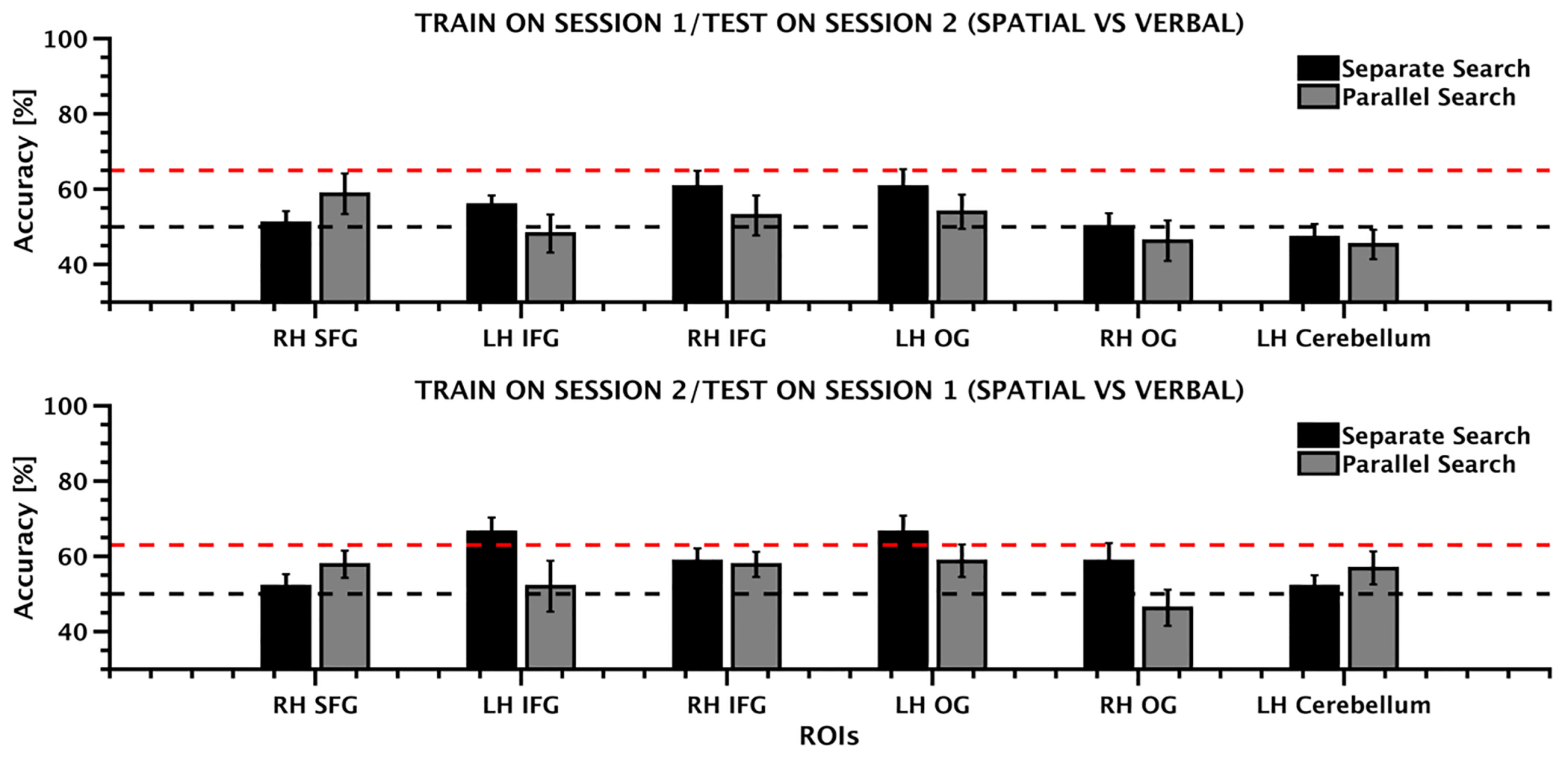
ROI MVPA inter session results. Mean classification accuracy for the two types of search (separate, parallel) shown separately for each ROI when the classifier was trained on session 1 and tested on session 2 (upper panel) and when it was trained on session 2 and tested on session 1 (lower panel). Error bars depict ±SEM (n = 13). The black line indicates the chance level (50%) while the red dashed line indicates the decoding level that is significantly above chance (>95^th^ percentile) derived by permutation testing.

Next, we tested whether the representation of domain was stable across sessions. This analysis revealed that the representation of domain could not be reliably decoded across the sessions. Though training the classifier on the first session and testing it on the second yielded above 50% accuracy in the SFG, bilateral IFG and left orbital gyrus, none of the regions showed accuracy above the 95^th^ percentile. A similar pattern of results was observed when the classifier was trained on the second session and tested on the first one, again with no region surpassing the 95^th^ percentile. The results are shown in Fig 6 and Table 4 reports the classification performance for each ROI and analysis direction.

**Table 4:** The mean and the standard error mean of the classification performance are reported separately for each ROI, for each direction and for each search type. The 95^th^ percentile calculated from the permutation testing for each direction is reported in parentheses.

	Train on Session 1/Test on Session 2 (95^th^: 64%)		Train on Session 2/Test on Session 1 (95^th^: 64%)	
	Separate Search	Parallel Search	Separate Search	Parallel Search
R SFG	50.96±3.30	58.65±5.36	51.92±3.42	57.69±3.61
L IFG	55.76±2.69	48.07±5.07	66.34±4.09	51.92±6.67
R IFG	60.57±4.87	53.84±4.56	66.34±4.56	58.65±4.33
L OG	60.57±4.44	52.88±5.32	58.65±3.57	57.69±3.33
R OG	50.00±3.74	46.15±5.36	58.65±4.98	46.15±4.77
L Cer	47.11±3.78	45.19±3.88	51.92±3.11	56.73±4.38

**Fig 7:**
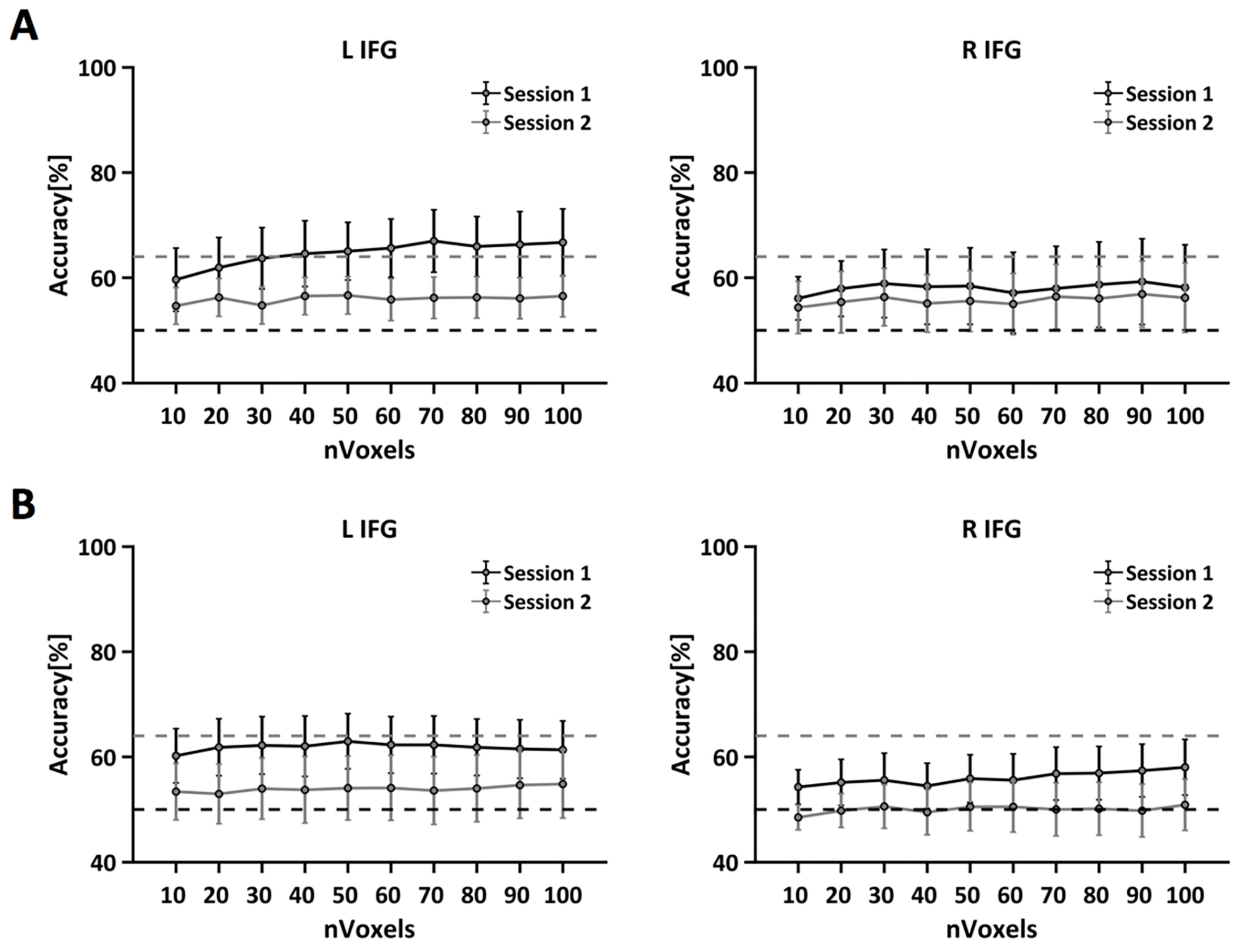
Decoding of domain during separate (A) and parallel (B) search is shown as a function of the number of voxels included in the analysis for each session. Also shown are chance decoding performance (black line) and the performance level that is significantly above chance (>95^th^ percentile) derived from permutation testing (grey dashed line).

Finally, we tested the ability of the SVM to classify domain as a function of different subsets of voxels. Fig 7 shows the results separately for the left and the right IFG and for each search type (separate and parallel).

If a cortical region is sensitive to domain, then the decoding accuracy is expected to rapidly increase as a function of the number of features included in the analysis, and then stabilize at high levels. This pattern is evident for the left IFG in the separate search condition. In this case, the decoding accuracy reached 66.72% when the classifier was run on the first session, indicating a reasonable level of sensitivity to rule domain. On the other hand, this pattern is absent in the right IFG where the classifier performance is either at chance level or below the chosen significance level.

The pattern of results for the parallel search condition is similar to those reported above. In the first session, the highest classification accuracy is still observed in the left IFG, although the decoding performance (62.96%) does not reach the criterion for statistical significance (which was 65%). In all the right IFG, the classification performance is steady at chance level. In the second session, both the regions show accuracies below the criterion for significance, indicating that the classifier was unable to distinguish between the verbal and spatial domains for the parallel search.

## Discussion

Previous studies have shown that the left IFG is sensitive to inductive reasoning applied to both the spatial and verbal domains [12,16]. In the current study we sought to assess whether the left IFG encodes information about rule domain as a way to understand the processes computed by this region. The results suggested that left IFG encodes domain-specific information. This result was clear in the first session and suggested by the pattern of results obtained in the second session, though the specific representations were not stable between the two sessions.

### The representation of rule domain

At least part of the information needed to represent rule domain was successfully decoded in the left IFG. On one side, this result suggests that both spatial and verbal types of information could be available in this region in the form of neural population codes [33]. On the other side, however, this result suggests that the information decoded by the classifier could possibly be influenced by different cognitive trajectories captured by our HRF model. The current experiment does not allow us to rule out either of these interpretations. Further experiments can try to address this riddle.

These interpretations could however hold only for the separate search condition. In the parallel search condition, the decoding accuracy failed to reach the 95^th^ percentile threshold in the left IFG. As participants needed to search both domains in this condition, the left IFG was likely processing information belonging to both the verbal and spatial domains concurrently, resulting in the two neural codes competing for resources (e.g., metabolic resources). This competition likely resulted in a noisier neural code, which would ultimately deteriorate the representation of rule domain during this condition. A complementary reason could be that, under a parallel rule search condition, to have overlapping neuronal populations coding for both domains in this brain region could have been instrumental for the specific nature of this task.

The rule domain was apparently decodable in the left OG, where the decoding ability in distinguishing the two domains during the parallel search reached the significance level. However, this higher accuracy could be generated by the difference between the two domains in terms of beta values. Additionally, rule domain was not decodable at the 95^th^ percentile threshold in any of the other brain regions involved in inductive reasoning (right IFG, right OG, right SFG, left cerebellum).

We further focused on the specific role of the left and the right IFG. Although both of these regions are known to be involved in inductive reasoning, we were able to decode rule domain only in the left IFG. This effect could be due to the fact that the size of the left IFG is larger than the size of the right IFG, and therefore the classification accuracy could have been inflated in the former region. To assess this possibility we directly tested the effect of the number of voxels considered on the classifier accuracy in the left and in the right IFG. These analyses again revealed that rule domain was decodable in the left but not in the right IFG. This result suggests that the neural population code underlying the spatial and verbal domains in the left IFG is genuine and was not an artifact of the difference in the number of voxels across regions.

The finding that rule domain is represented in the left IFG, but not other regions involved in inductive reasoning, seems particularly robust as the data from the second session yielded remarkably similar results. In those data, rule domain was successfully decoded in the left IFG under the separate search condition and using a limited subset of voxels, while no other conditions or regions resulted in above threshold decoding accuracy. This replication suggests that the left IFG is reliably recruited to compute domain-specific processes during inductive reasoning.

### The left IFG and its role in inductive reasoning

These results support a crucial role of left IFG during the implementation of higher cognitive functions and, more specifically, inductive reasoning. The involvement of the left IFG we observed is consistent with previous work on inductive reasoning [34–37] as well as with previous studies that attempted to explore the relationship between IFG and executive functions by means of multivariate analysis [38–40].

This explanation would be in line with previously reported evidence [41] in favor of the ‘sculpting the response space’ hypothesis [42], which sees the left dorso-lateral prefrontal cortex as crucial for defining a set of responses suitable to accomplish a task. This interpretation is also consistent with the hypothesis suggesting that the left hemisphere could work as an ‘interpreter’ [43,44]. This theory views the left hemisphere as a system that processes bits of incomplete information and connects them into clear patterns, which are then used to make sense of the world. Connecting information is a process that relies on forming meaningful associations between items [45]. Fletcher and colleagues (50) measured the hemodynamic activity associated with the process of learning new semantic relationships when previous relationships with the same stimuli had already been created. Their results showed that the act of forming meaningful associations produced an increase in the hemodynamic activity in the left PFC. Forming this kind of association is an essential part of inductive reasoning.

On the whole, we think that the left IFG might hold a population of neurons tuned to process verbal information, while the right IFG might hold a different population of neurons tuned to process spatial information, as shown by our previous study [16]. Additionally, we suggest that the information processed by the domain-dependent neurons located in the left (verbal) and in the right IFG (spatial) could be integrated by a subset of neurons which flexibly code for whatever domain is task-relevant, located in the left IFG, an explanation supported by the successful decoding of rule domain in the left but not in the right IFG, as shown in this study. Overall, this interpretation suggests that inductive reasoning would be localized in the left PFC but not in the right PFC, and would be a domain-independent process, although new studies are necessary to further corroborate our interpretation.

Particularly interesting is the study by Baggio and colleagues [40], in which participants were asked to complete a task that relied on logical connectives, such as ‘and’, ‘or’ and ‘if.’ Specifically, the participants were first presented with visual cues representing specific combinations of logical compound (e.g., “there is a yellow square and a green circle”), which was then followed by a visual scene made of geometrical figures (e.g., a yellow square close by a green circle). The participants had to judge whether the visual scene matched the combination of logical compounds. The authors were able to decode information about the logical connectives in the left IFG. This finding is interesting as evaluating information about logical connectives was crucial for the reasoning task to be completed, in the same way as evaluating information about rule domain was crucial to completing the reasoning task presented in our study.

### Dynamic recruitment

Our results also show that the classifier was unable to decode rule domain across the two sessions, despite being reliably decoded within each session. On one side, this discrepancy could be partially explained by subtle differences in the procedures used to acquire and analyze the data, such as tiny differences in the scanner parameters, or small misalignments in the co-registration. On the other side, this result could be a genuine effect, suggesting that the recruitment of the left IFG during inductive reasoning could be a process that dynamically employs different neuronal sub-populations. The representations of domain created in the first session likely faded over the two years, as they were not being used, and then new representations were formed in the same region at the second session. Thus, it seems that the neurons in left IFG are dynamically recruited to load the representation of the rules at hand, though these representations may change over time. Dynamic recruitment of the prefrontal regions is evidence also by the recruitment of the same regions by similar, but non-overlapping, tasks, such in the study by Baggio and colleagues (59).

The neural mechanisms underlying such dynamic recruitment are still under investigation. Our explanation is that different populations of neurons within the same brain region may carry out different operations. This explanation is consistent with the study by Wendelken and colleagues [46], in which the authors reported that visuo-spatial and semantic information both produced a considerable activation within the bilateral rostrolateral PFC (rlPFC) and that the two peaks of activations were located in different parts of the left rlPFC. Wendelken and colleagues suggest that rlPFC neurons process domain general information, but specific populations of neurons could process domain-specific information, and we hypothesize that a similar mechanism could explain our results as well. Although our results are in line with what was suggested by Wendelken and colleagues (60), it is important to note that the brain region we are focusing on in this study (left IFG) is different from the one identified by Wendelken (left rlPFC). This difference in terms of brain regions could suggest that the mechanism described above could be a general one, shared by more than one set of prefrontal neurons.

### Representations of processes and representation of stimuli

The inferences drawn so far must be taken with prudence. In fact, the success in classifying the two patterns underlying the verbal and the spatial domain could arise from or be affected by different underlying cognitive processes. For example, inferring patterns is a complex process that most likely requires participants to perform multiple tasks at the same time. Probably they were viewing the stimulus, reading letters, generating hypotheses, and possibly validating their hypotheses. Most of these processes might be shared between the two domains, and yet some of these processes could be recruited more intensely in one domain than in the other. For instance, when participants were told that the rule domain was verbal, then they could have accessed the lexical system to make sense of the letters read. Accessing the lexical system could happen more frequently when the rule domain is verbal than when is spatial. As a result, the classifier could have picked up this subtle difference and use it to distinguish the representation of rule domain. However, such subtle differences could also be the necessary characteristic that defines representations of cognitive processes. Specifically, the representation of each rule domain could be defined by a particular configuration of cognitive processes that are engaged at the same time, in the same way the representation of a human face could be defined by a particular configuration of physical attributes forming the image. This is an important difference to keep in mind when we discuss representations of processes (e.g., rule domain) instead of representations of stimuli (e.g., face). In summary, if our classifier picked up subtle differences in terms of cognitive processes related to rule discovery, and if representations of rule domain are defined by specific configurations of cognitive processes, then we could safely conclude that what we decoded was in fact the representation of rule domain.

A possible limitation of the present study was the relatively small sample size. The reason why we focused on 13 participants only was to assess reliability and stability of multi-voxel pattern representations through a longitudinal study, in which these 13 participants were available for a second fMRI session two years after the first session. Nevertheless, we were still able to show successful classifications in selected brain regions and executive functions have been successfully examined with similar sample sizes in previous studies [47] [48].

In conclusion, the present data support the hypothesis that frontally-based hemispheric specialization could be process-based [9]. In particular, our data suggest that the left vlPFC contains populations of neurons specifically tuned to process domain-dependent information required to carry out inductive reasoning tasks, by creating domain-based rule representations that may dynamically change across sessions. Furthermore, in line with previous literature [10], we suggest that hemispheric asymmetries may vary as a function of the difficulty and complexity of the inductive reasoning task, with the right hemisphere working as a complementary system with respect to the left hemisphere.

The reason for this result could be that the left IFG reaches its full processing capacity, an explanation which is consistent with previous evidence suggesting that a load-sensitive bilateral fronto-parietal network may be engaged whenever a hemisphere has reached its limit in learning and executing new rules [49,50]. The slightly higher engagement of the right IFG observed when participants had to keep looking for both domains could also result from the right hemisphere limiting the number of possible patterns.

In fact, searching for both verbal and spatial domains requires participants to evaluate verbal patterns as well as spatial patterns. In order to optimize the search process, the right IFG could constrain the possible number of patterns to evaluate.

This inter-hemispheric asymmetry could arise particularly when the cognitive demands imposed by the inductive reasoning task are fairly low, such as when the rule domain to attend to is known (i.e., separate rule search). In fact, when the cognitive demands are low, the computational capacity of a single hemisphere could suffice to successfully execute the task at hand [49]. However, when the task demands increase, such as when the rule domain to attend to is unknown (i.e., parallel rule search), the left IFG could reach its computational limit. As a result, part of the resources needed to integrate the domain-specific information could be allocated on the right IFG, which would therefore support the left IFG in order to find relationships among the available information.

## Supporting information

S1 Appendix (PDF)
