# Supplementary material for: Decoding rule search domain in the left inferior frontal gyrus
Source: PLoS One. 2018 Mar 16;13(3):e0194054. doi: 10.1371/journal.pone.0194054 (PMC5856266; doi:10.1371/journal.pone.0194054)
Supplement: S1 Appendix — (PDF) [file pone.0194054.s001.pdf]

Appendix: The 40 experimental pattern categories (From Babcock and Vallesi, 2015)

Category Group	*Category Domain*	*Category Description*	*Category Exemplars*
A	Spatial	Horizontal zig-zag	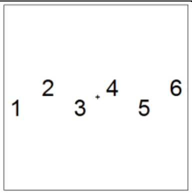
A	Spatial	N shape	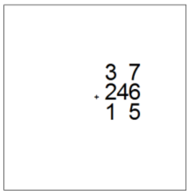
A	Spatial	Horizontal line wrapped around the square, ending at the starting point	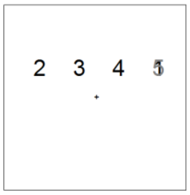
A	Spatial	U shape	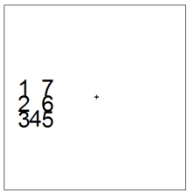
A	Spatial	Bottom-left to top-right diagonal line	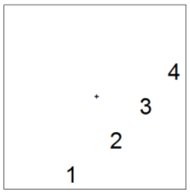
A	Spatial	Right triangle	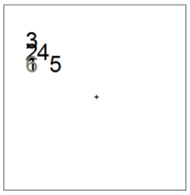
A	Spatial	Vertical line with the distance between items doubling until the edge of the square	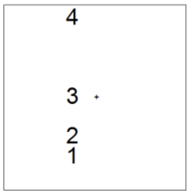

A	Spatial	Y shape	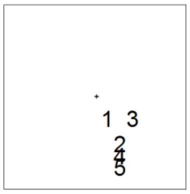
A	Spatial	Circle around center fixation cross	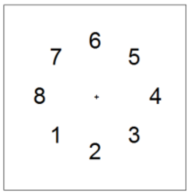
A	Spatial	2-6 items close together in a row then 2 items close together somewhere else	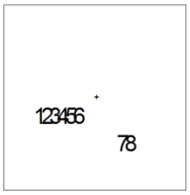
A	Verbal	Types of sports	golf-golf, vela-sailing, corsa-running, lotta-wrestling, ippica-horseracing, tennis-tennis, pattino-skating, scacchi-chess, biliardo-pool, ciclismo-cycling
A	Verbal	City places	faro-lighthouse, molo-pier, banca-bank, duomo-cathedral, piazza-square, stadio-stadium, mercato-market, palazzo-palace, farmacia-farmacy, stazione-station
A	Verbal	Fruits	mora-blackberry, pera-pear, pesca-peach, cedro-citron, ananas-pineapple, prugna-prune, fragola-strawberry, lampone-raspberry, ciliegia-cherry, cocomero-watermelon
A	Verbal	Astronomy	luna-moon, nova-nova, giove-Jupiter, urano-Uranus, cometa-comet, nebula-nebula, meteora-meteor, saturno-Saturn, galassia-galaxy, mercurio-Mercury
A	Verbal	Art forms	film-movie, foto-photo, ballo-dance, opera-opera, design-design, quadro-painting, canzone-song, mosaic-mosaic, ritratto-portrait, scultura-sculpture
A	Verbal	Sea animals	foca-seal, orca-killer whale, manta-mantaray, polpo-octopus, medusa-jellyfish, squalo-shark, delfino-dolphin, ostrica-oyster, aragosta-lobster, tricheco-walrus
A	Verbal	Tools	pala-shovel, sega-saw, metro-tape measure, pinza-pliers, cesoia-shears,

			chiodo-nail, forcone-pitchfork, trapano-drill, giravite-screwdriver, martello-hammer
A	Verbal	Toys	dado-dice, lego-legos, corda-jump rope, robot-robot, biglia-marble, puzzle-puzzle, bambola-doll, peluche-stuffed animal, aquiline-kite, trottola-spinning top
A	Verbal	Methods of transportation	nave-ship, tram-tram, barca-boat, treno-train, camion-truck, camper-camper, funivia-cableway, gondola-gondola, roulette-camping trailer, trattore-tractor
A	Verbal	Clothing items	polo-polo shirt, tuta-tracksuit, felpa-sweatshirt, gonna-skirt, giacca-jacket, scarpa-shoe, cintura-belt, stivale-boot, cappello-hat, giubbino-denim jacket
B	Spatial	T shape	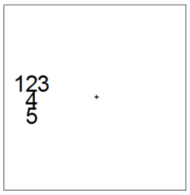
B	Spatial	Semi-circle around center fixation	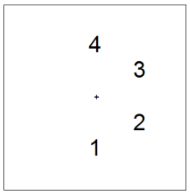
B	Spatial	L shape	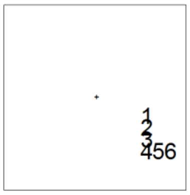
B	Spatial	Staircase pattern to the left or right edge	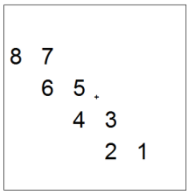
B	Spatial	Horizontal line with the distance between items doubling until the edge of the square	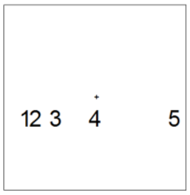

B	Spatial	Regular polygon with the final item in the center of the polygon	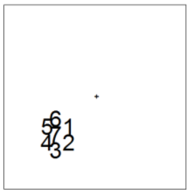
B	Spatial	Vertical zig-zag	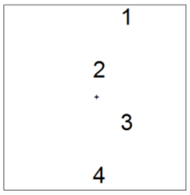
B	Spatial	Vertical line wrapped around the square, ending at the starting point	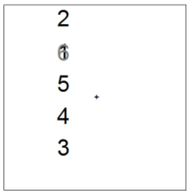
B	Spatial	W shape	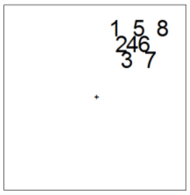
B	Spatial	Narrowing triangle	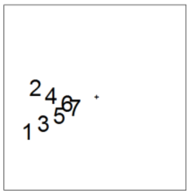
B	Verbal	Musical instruments	arpa-harp, oboe-oboe, basso-bass, liuto-lute, flauto-flute, organo-organ, fagotto-bassoon, tamburo-drum, armonica-harmonica, chitarra-guitar
B	Verbal	School items	biro-pen, riga-ruler, gomma-eraser, zaino-backpack, fodera-slipcover, matita-pencil, appunti-notes, lavagna-blackboard, astuccio-pencil case, quaderno-notebook
B	Verbal	Beverages	coca-coke, vino-wine, latte-milk, succo-juice, aperol-aperol, grappa-grappa, gassosa-sprite, liquore-liquor, prosecco-prosecco, spremuta-fresh-squeezed juice
B	Verbal	Sports equipment	arco-bow, remo-oar, mazza-bat, sella-saddle, pagaia-paddle, volano-shuttlecock, birillo-bowling pin, frisbee-frisbee, canestro-basket (for basketball), manubrio-barbell
B	Verbal	Materials	lana-wool, seta-silk, marmot-marble,

			vetro-glass, cotone-cotton, pietra-stone, mattone-brick, argilla-clay, acrilico-acrylic, carbonio-carbon
B	Verbal	Parts of the face	naso-nose, gota-cheek, barba-beard, mento-chin, ciglia-eyelash, labbro-lip, capelli-hair, basetta-sideburn, fossetta-dimple, palpebra-eyelid
B	Verbal	Vegetables	fava-broad bean, rapa-turnip, aglio-garlic, porro-leek, cavolo-cabbage, rucola-arugula, cicoria-chicory, spinaci-spinach, asparago-asparagus, peperone-pepper
B	Verbal	Mammals	lupo-wolf, toro-bull, capra-goat, leone-lion, pecora-sheep, riccio-hedgehog, cavallo-horse, donnola-weasel, coniglio-rabbit, marmotta-marmot
B	Verbal	Weather phenomena	gelo-frost, neve-snow, tuono-thunder, vento-wind, nebbia-fog, tifone-typhoon, tornado-tornado, uragano-hurricane, grandine-hail, tempesta-storm
B	Verbal	Items found in the bathroom	bide-bidet, file-floss, luffa-loofah, vacsa-tub, doccia-shower, rasoio-razor, balsamo-conditioner, pettine-comb, pinzette-tweezers, toilette-toilet

Notes: For spatial categories, the category exemplars column shows one possible exemplar from the category. Numbers indicate the order of item presentation, in the actual task these would be replaced by randomly selected letters. For the verbal categories, the category exemplars column shows the ten possible Italian words for each category, followed by an English translation.
